# *Fusimonas intestini* gen. nov., sp. nov., a novel intestinal bacterium of the family *Lachnospiraceae* associated with diabetes in mice

**DOI:** 10.1038/s41598-017-18122-2

**Published:** 2017-12-22

**Authors:** Hiroyuki Kusada, Keishi Kameyama, Xian-Ying Meng, Yoichi Kamagata, Hideyuki Tamaki

**Affiliations:** 1 Bioproduction Research Institute, National Institute of Advanced Industrial Science and Technology (AIST), Central 6, 1-1-1 Higashi, Tsukuba, Ibaraki 305-8566 Japan; 2 Frontier Research Laboratories, Institute for Innovation, Ajinomoto Co., Inc., 1-1 Suzuki-cho, Kawasaki, Kanagawa 210-8681 Japan

## Abstract

Our previous study shows that an anaerobic intestinal bacterium strain AJ110941^P^ contributes to type 2 diabetes development in mice. Here we phylogenetically and physiologically characterized this unique mouse gut bacterium. The 16S rRNA gene analysis revealed that the strain belongs to the family *Lachnospiraceae* but shows low sequence similarities ( < 92.5%) to valid species, and rather formed a distinct cluster with uncultured mouse gut bacteria clones. In metagenomic database survey, the 16S sequence of AJ110941^P^ also matched with mouse gut-derived datasets (56% of total datasets) with > 99% similarity, suggesting that AJ110941^P^-related bacteria mainly reside in mouse digestive tracts. Strain AJ110941^P^ shared common physiological traits (e.g., Gram-positive, anaerobic, mesophilic, and fermentative growth with carbohydrates) with relative species of the *Lachnospiraceae*. Notably, the biofilm-forming capacity was found in both AJ110941^P^ and relative species. However, AJ110941^P^ possessed far more strong ability to produce biofilm than relative species and formed unique structure of extracellular polymeric substances. Furthermore, AJ110941^P^ cells are markedly long fusiform-shaped rods (9.0–62.5 µm) with multiple flagella that have never been observed in any other *Lachnospiraceae* members. Based on the phenotypic and phylogenetic features, we propose a new genus and species, *Fusimonas intestini* gen. nov., sp. nov. for strain AJ110941^P^ (FERM BP-11443).

## Introduction

The mammalian digestive tract is one of the largest microbial habitats: more than 10^14^ cells of microorganisms are present in the entire human gastrointestinal tract^1^, and anaerobic bacteria are the main constituents of the ecosystems^2^. Recent extensive studies based on next generation sequencing approach enabled to characterize composition, diversity, and spatial distribution of gut microbial communities^3^, and suggested that changes in the gut microbiota might be associated with human health and diseases. This attracts a worldwide interest for its potential impact in the field of medical science as well as microbial ecology^4^.

Culture-independent metagenomic studies revealed that members of the phyla *Bacteroidetes* and *Firmicutes* represent the most dominant and prevalent bacterial groups in the human gut ecosystem, and the *Firmicutes* is in particular the main component accounting for >50% of all the 16S rRNA gene sequences^5–7^. These intestinal *Firmicutes* bacteria are known to have an influence on the human health. For instance, several *Lactobacillus* spp. in the family *Lactobacillaceae* are widely recognized as beneficial bacteria (lactic acid bacteria) for maintaining the intestinal environment^8^, whereas some *Clostridium* spp. in the family *Clostridiaceae* are known as pathogens causing a bowel inflammation and food poisoning^9^.

Members of the family *Lachnospiraceae* constitute the abundant taxa within the phylum *Firmicutes* in human gut microbiota^7^. Over half of human intestinal bacteria are not cultivated yet and many of those belong to the family *Lachnospiraceae* ^10^. Recently, new bacterial strains representing novel genera in the family *Lachnospiraceae* were isolated from not only digestive tract but also microbial mat and biogas reactor, *i*.*e*., *Eisenbergiella tayi* strain B086562^T11^, *Fusicatenibacter saccharivorans* strain HT03–11^T^ ^12^, *Herbinix hemicellulosilytica* strain T3/55^T^ ^13^, *Mobilitalea sibirica* strain P3M-3^T^ ^14^, *Murimonas intestini* strain SRB-530-5-H^T^ ^15^ and *Anaerobium acetethylicum* GluBS11^T^ ^16^. The family *Lachnospiraceae* currently consists of 31 genera according to the LPSN database (http://www.bacterio.net/-news.html), Bergey’s Manual of Systematic Bacteriology (second edition), and recent study by Patil *et al*.^16^.

Importantly, based on metagenomics, Qin *et al*. reported that the abundance of *Lachnospiraceae* bacteria in the gut was positively correlated with type 2 diabetes, one of the major public health concerns^17^, implying that the *Lachnospiraceae* might be associated with occurrence of the disease. However, no clear evidence was shown due to the lack of axenic cultures of the possible causing agent. Very recently, we succeeded in isolating a new member of the family *Lachnospiraceae*, designated strain AJ110941^P^, from the feces of hyperglycemic obese mouse^18^, and further demonstrated that the new isolate is obviously involved in development of obesity and diabetes in germ-free (GF) *ob/ob* mice^18^. In fact, the intestinal colonization of strain AJ110941^P^ in GF mice induced the typical symptoms such as significant increases in fasting blood glucose levels together with liver and mesenteric adipose tissue weights, and decrease in plasma insulin levels and HOMA-β values^18^. In this work, we phylogenetically and physiologically characterized the new strain AJ110941^P^, compared its phenotypic characteristics with other *Lachnospiraceae* species, and consequently propose the novel genus and species, *Fusimonas intestini* gen. nov., sp. nov., for this strain.

## Results and Discussion

### Phylogenetic affiliation of strain AJ110941^P^ and its closest relatives

**Figure 1:**
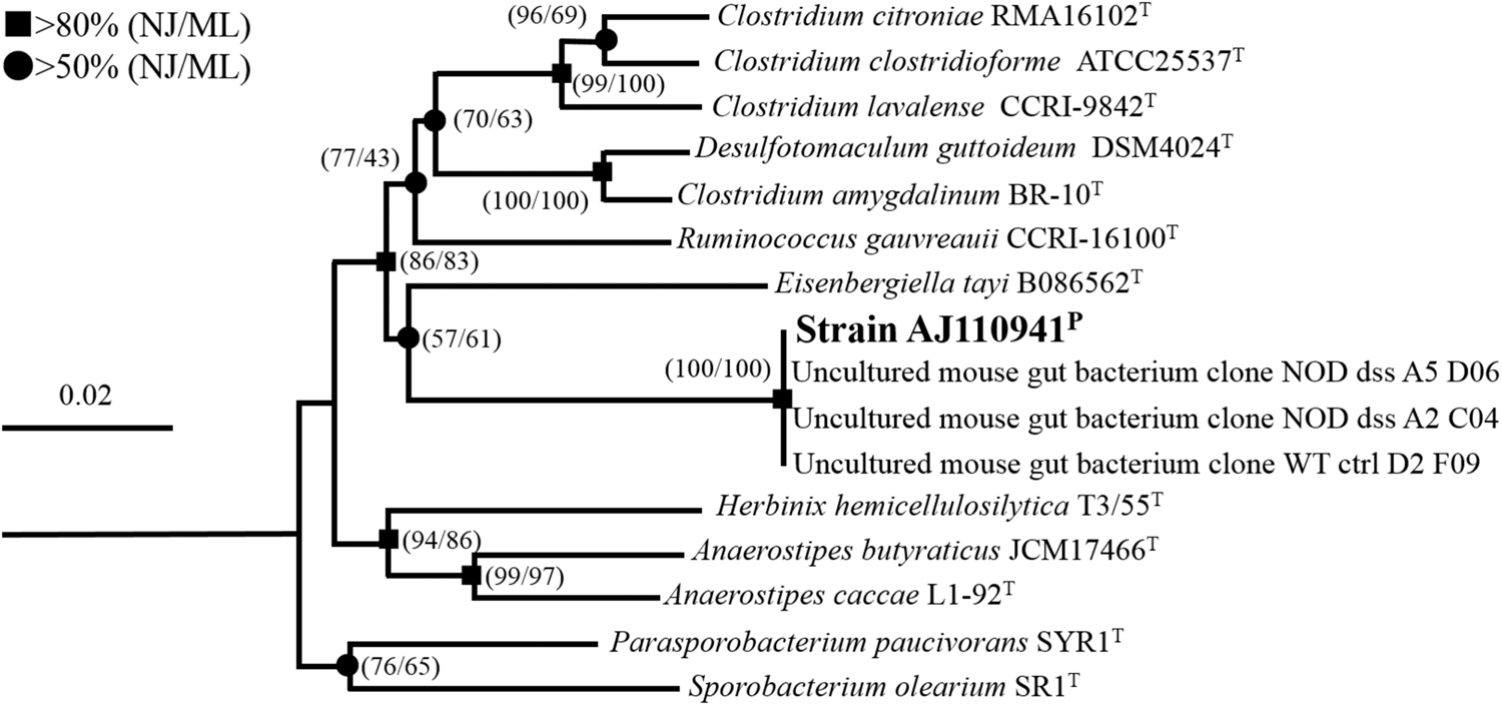
Phylogenetic relationships between strain AJ110941^P^ and the closely related members of the family *Lachnospiraceae* based on 16S rRNA gene sequences. The phylogenetic tree was constructed by neighbor-joining (NJ) method. The 16S rRNA gene sequence of *Peptostreptococcus anaerobius* JCM1470^T^ (AB640688) was used as an outgroup. Bootstrap values of >50% and >80% estimated using neighbour-joining (NJ) and maximum-likelihood (ML) methods (1,000 replications) are shown by circle and square at branching points, respectively.

**Figure 2:**
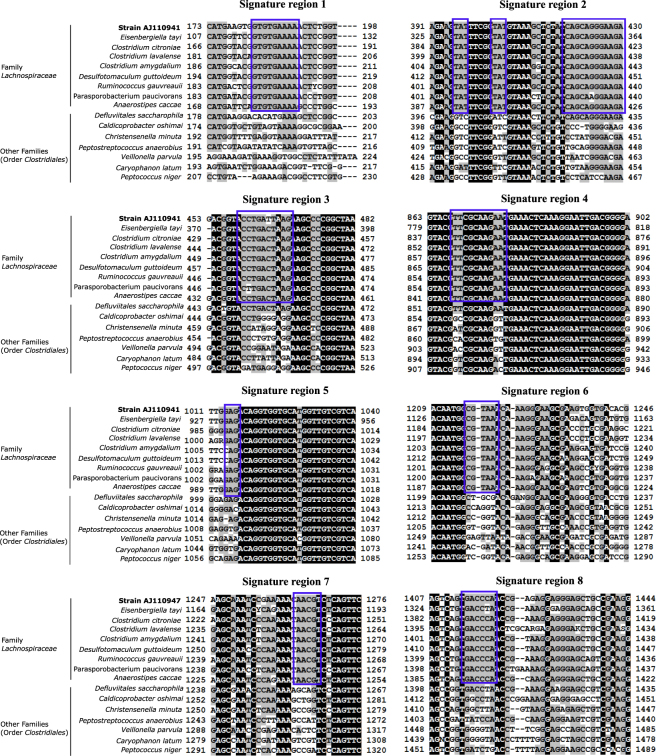
Alignment of 16S rRNA gene sequences within signature regions able to distinguish between *Lachnospiraceae* species and other family members. The 16S rRNA genes of *Lachnospiraceae* species were aligned with corresponding sequences from microorganisms belonging to the order *Clostridiales*. Identical and similar nucleotides are indicated by black and gray backgrounds, respectively. Signature sequences are boxed in blue.

Comparison of 16S rRNA gene sequence of strain AJ110941^P^ with those of validly described species indicated that the strain is moderately related to members of the family *Lachnospiraceae* with relatively low sequence similarities ( < 92.5%). The most closely related species to strain AJ110941^P^ was *Ruminococcus gauvreauii* strain CCRI-16110^T^ isolated from the human faecal specimen (92.5% sequence similarity)^19^. Other close relatives were *Clostridium amygdalinum* strain BR-10^T^ (92.3%)^20^, *Clostridium citroniae* strain RAM16102^T^ (92.1%)^21^, *Eisenbergiella tayi* strain B086562^T^ (92.1%)^11^, *Anaerostipes caccae* strain L1-92^T^ (92.0%)^22^, *Clostridium lavalense* strain CCRI-9842 (92.0%)^23^, and *Desulfotomaculum guttoideum* strain DSM 4024 ^T^ (92.0%)^24^. Strain AJ110941^P^ formed a monophyletic cluster with those relatives and was a neighbor of *Eisenbergiella tayi* strain B086562^T^ (Fig. 1). We further performed multiple alignment of 16S rRNA gene sequences, and found eight signature regions that are highly conserved only among *Lachnospiraceae* species, but not in other families of the order *Clostridiales* (Fig. 2). These results suggest that strain AJ110941^P^ is affiliated with the family *Lachnospiraceae*, and that a new genus should be created for the novel strain because of its low sequence similarities (92.5%) to the close relatives.

Comparative 16S rRNA gene sequence analysis revealed that strain AJ110941^P^ showed high 16S rRNA gene sequence similarities (99–100%) to clones retrieved from the mouse digestive tracts: uncultured bacterial clones WT ctrl D2 F09, NOD dss A5 D06 and NOD dss A2 C04 from mice colon tissues^25^, clones 16saw23-2g06.p1k and 16saw23-1b01.p1k from mice cecal contents^26^, clone F09 from wild-type mouse’s colon tissue, clones D06 and C04 from colon tissue samples from Nod2 KO mice^25^, and clones 16saw23-2g06.p1k and 16saw23-1b01.p1k from avirulent pathogen infected mouse^26^. Based on the IMNGS^27^ search (metagenome-derived 16S rRNA gene sequence database search), we further found that the 16S rRNA gene sequence of strain AJ110941^P^ matched with 3699 and 826 datasets (with >97% and >99% similarity, respectively), 56% of which were derived from mouse guts (Supplementary Fig. S1). These results suggest that *F*. *intestini* and its relatives are dwelling mainly in mouse gastrointestinal tracts.

### Morphological, physiological, and biochemical characteristics

**Figure 3:**
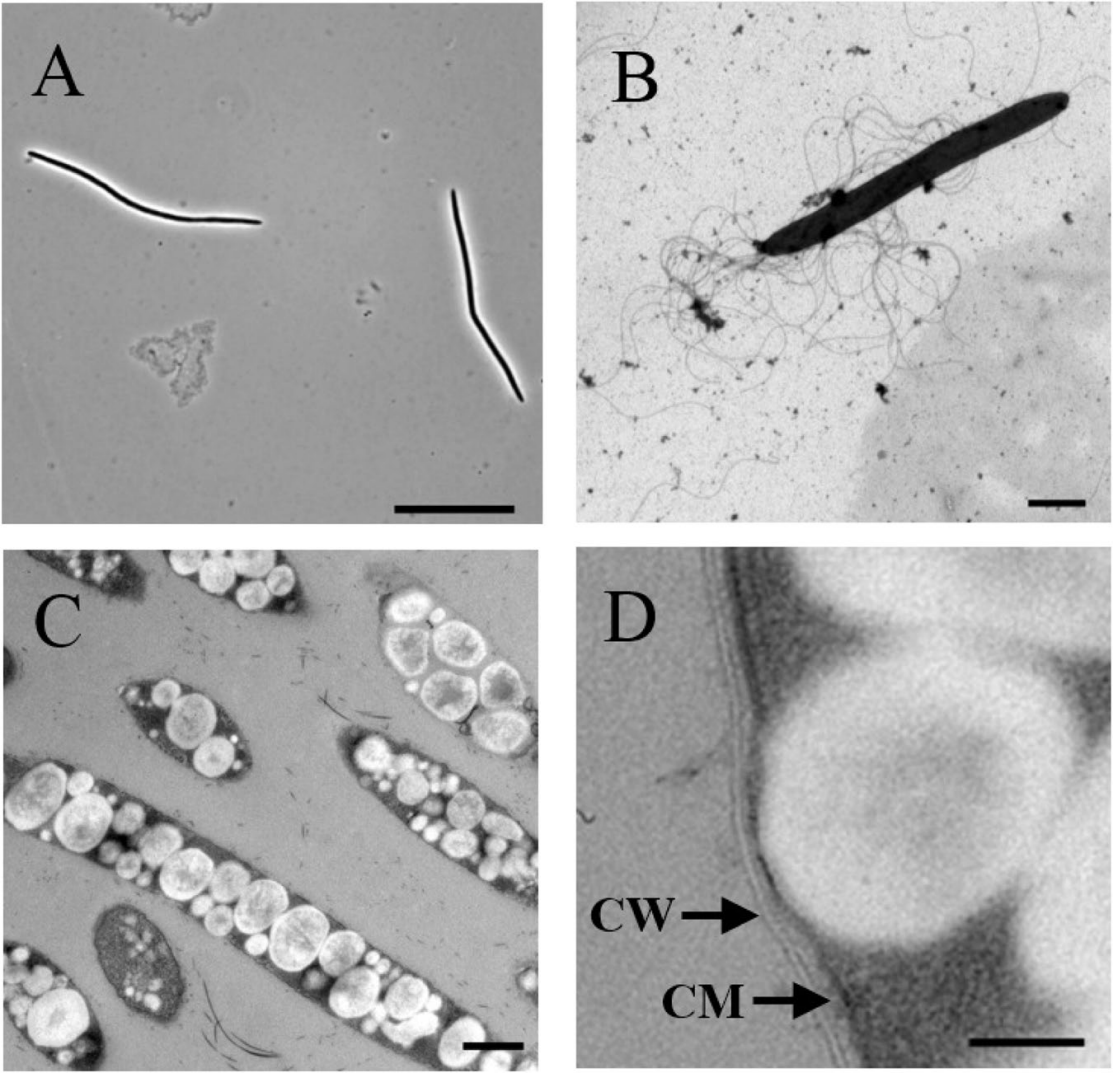
Photomicrographs of strain AJ110941P grown in GAM medium under anaerobic conditions at 37 °C. (**A**) Phase-contrast photomicrograph. (**B**) Transmission electron micrograph of negatively stained cells. (**C**) Transmission electron micrograph of polyhydroxyalkanoate-like compounds accumulated in the cells. (**D**) Ultrathin section showing the Gram-positive cell wall (CW) and the cytoplasmic membrane (CM). Bars, 20 μm (A), 2 μm (**B**), 500 nm (**C**), and 100 nm (**D**).

Strain AJ110941^P^ was a strictly anaerobic and heterotrophic bacterium. The temperature range for growth of strain AJ110941^P^ was 15–40 °C (optimum growth at 37 °C). No growth was observed at 10 °C and 45 °C. The strain grew at pH 6.5–8.0, with an optimum at pH 8.0, and no growth occurred at pH 6.0 and 8.5. The strain did not require NaCl for growth and tolerated up to 0.5% (w/v) NaCl. Brown disk-like colonies (0.1–1.4 mm in diameter and <0.2 mm height) were formed in the GAM agar medium after 4 days of incubation at 37 °C. Cells were fusiform-shaped rods, 9.0–62.5 µm in length and 0.55–0.9 µm in width (mean size 25.05 × 0.63 µm), which occurred mainly as single cells (Fig. 3A). Spore formation was not observed. Cells possessed multiple flagella (Fig. 3B), but the motility was not observed. Intracellular polyhydroxyalkanoate (PHA) like compounds were observed (Fig. 3C). Cells were positively stained by Gram-staining and also showed Gram-positive type of cell wall by electron microscopy (Fig. 3D).

The biochemical tests indicated that strain AJ110941^P^ showed positive enzymatic activity for naphthol-AS-BI-phosphohydrolase, α-galactosidase, β-glucuronidase, β-glucosidase, α-glucosidase, α-arabinosidase and *N*-acetyl-β-D-glucosaminidase, whereas negative reactions were obtained for alkaline phosphatase, esterase, lipase, arylamidase, trypsin, chymotrypsin, acid phosphatase, β-galactosidase, α-mannosidase, α-fucosidase, urease, arginine dihydrolase, glutamate decarboxylase, and protease. Catalase reaction was negative. The main end-products of glucose fermentation were short chain fatty acids such as acetate, butyrate, and lactate, and carbon dioxide. The carbon source utilization test revealed that strain AJ110941^P^ was able to utilize the following substrates (all at 20 mM, unless shown otherwise): glucose, lactose, maltose, raffinose, xylose, sucrose, trehalose (0.1%), cellobiose (0.1%), galactose, xylan (5 g l^−1^), starch (5 g l^−1^) and melibiose as a sole carbon source. The following substrates (all at 20 mM, unless shown otherwise) were not utilized: arabinose, rhamnose (0.1%), ribose, mannose, fructose, glycerol (5 mM), cellulose (0.1%), pectin (5 g l^−1^), soytone (5 g l^−1^), pyruvate, crotonate (10 mM), tryptone (0.1%), casamino acids (0.1%), yeast extract (0.1%), H_2_/CO_2_ (80:20, v/v, head space), inositol (10 g l^−1^), mannitol (0.1%), melezitose (0.1%) and sorbitol (0.1%). The results suggests that strain AJ110941^P^ prefers monosaccharides and polysaccharides as carbon source.

### Chemotaxonomic characteristics

Whole-cell fatty acid compositions, the major respiratory quinones, and G + C content of strain AJ110941^P^ were determined. The cellular fatty acid profiles were as follows: C_18:1_ *cis*9 (44.3%), C_16:0_ (22.5%), C_18:1_ *trans*9 or *cis*6 (11.4%), C_14:0_ (9.8%), C_18:0_ (8.5%), C_17:1_ or cycloC_17:0_ (3.5%). The major respiratory quinone was MK-5 (H4). The G + C content of genomic DNA was 41.1 mol%.

### Biofilm formation in strain AJ110941^P^ and relative species

**Figure 4:**
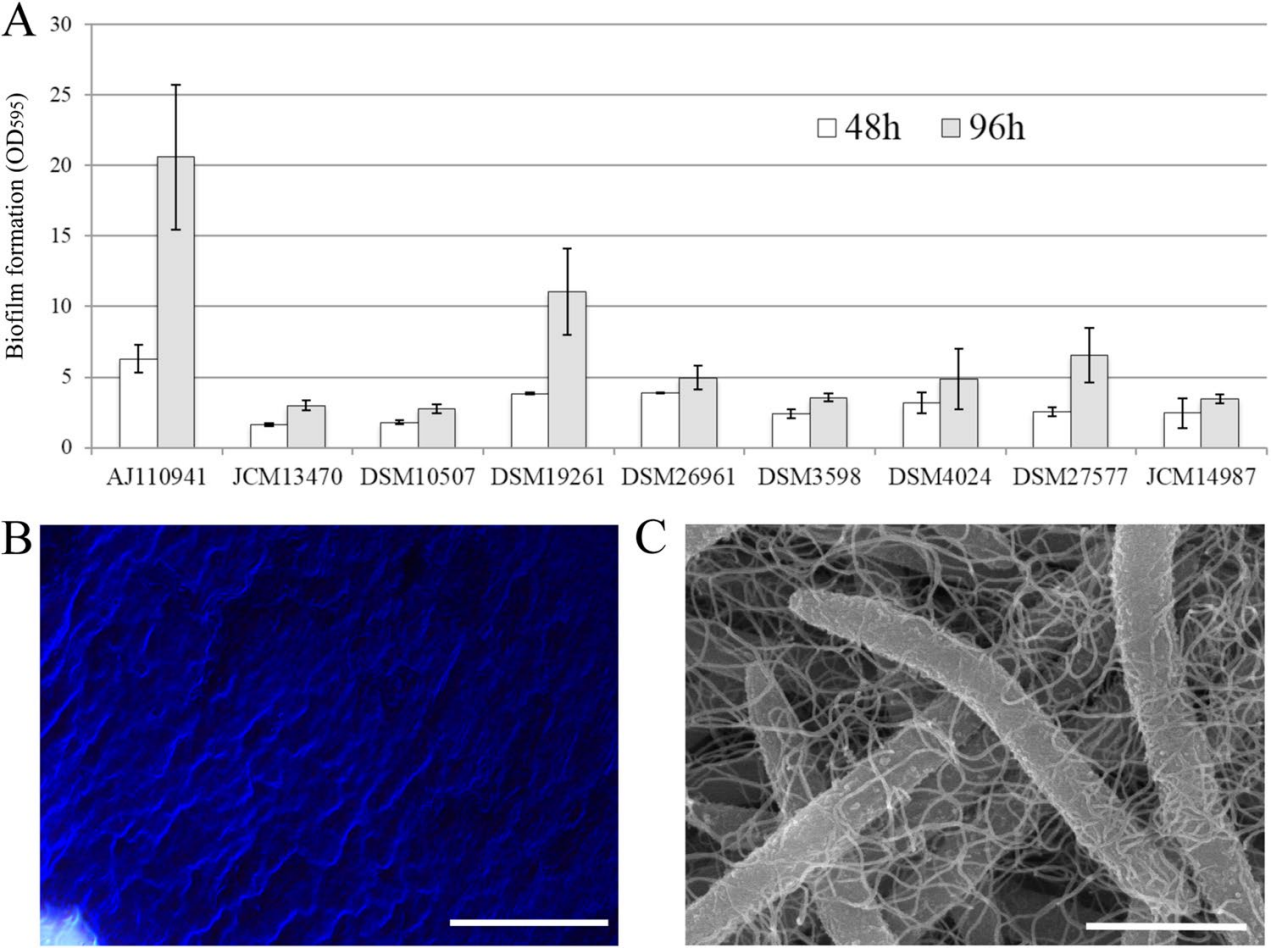
Comparison of biofilm forming-capacity among Lachnospiraceae strains. (**A**) Quantification of surface-attached biofilms of the *Lachnospiraceae* species grown in GAM medium at 37 °C for 48 h (white bars) and 96 h (gray bars), respectively. Biofilms were stained with crystal violet and quantified by measuring at 595 nm. The data represents the average of three biological replicates and the standard deviation is indicated by vertical bars. (**B**) Phase-contrast photomicrograph of biofilm-like aggregates of strain AJ110941^P^, after staining with crystal violet. (**C**) Scanning electron micrograph (SEM) of strain AJ110941^P^ grown in GAM medium at 37 °C for 48 h. Bars, 20 μm (B) and 1.5 μm (**C**).

Biofilm-forming capacity of strain AJ110941^P^ was determined by crystal violet (CV) method, and was compared with those of other *Lachnospiraceae* species. Strain AJ110941^P^ produced the highest amount of biofilm among the all *Lachnospiraceae* species tested (Fig. 4A). The microscopic analysis further showed that the CV-stained biofilm of strain AJ110941^P^ was rugged and polymerically-coated structure (Fig. 4B). Intriguingly, we also observed that strain AJ110941^P^ notably produced large amounts of extracellular polymeric substances (EPS) by scanning electron microscopy (Fig. 4C). Considering that EPS is widely known to play an important role in bacterial adhesion, both EPS-producing and biofilm-forming capacities of strain AJ110941^P^ might be associated with colonization in the mouse gastrointestinal tracts, though further investigation is required to verify this.

### Morphological and phenotypic comparisons among strain AJ110941^P^ and its close relatives

Morphological and phenotypic characteristics of AJ110941^P^ were compared with those of the close relatives within the family *Lachonospiraceae* showing more than 92% 16S rRNA gene sequence similarity to the novel strain (Table 1). The novel strain and the close relatives share several common physiological traits: it is a Gram-positive, mesophilic, anaerobic, and fermentative bacterium with relatively low G + C content. However, strain AJ110941^P^ possesses some unique features that differentiate it from other closely related species. One of the most distinctive features is its cell morphology. Strain AJ110941^P^ shows fusiform-shaped cells with obviously long length (9.0–62.5 μm), while all the other relatives are rod^11,21^, oval^20^ or coccus-shaped cells^19^ (0.5–10.0 μm in length). This new isolate also possesses multiple flagella (Fig. 3B), whereas other close relatives have no or only one terminal flagellum. Strain AJ110941^P^ contains C_18:1_ *cis*9 as the most abundant cellular fatty acid, but other relatives have C_16:0_. Furthermore, it can be distinguished from the other close relatives by its organic substrate utilization pattern. For example, lactose and raffinose can be utilized by only strain AJ110941^P^, whereas other substrates (*e*.*g*., glucose, maltose and sucrose) are commonly utilized. Besides, strain AJ110941^P^ produced large amounts of EPS, and showed the highest biofilm-forming capacity among *Lachnospiraceae* species tested in the present study (Fig. 4). By these clear distinctive phenotypic features and low 16S rRNA gene sequence similarities of less than 92.5%, strain AJ110941^P^ can be convincingly distinguished from the related genera within the family *Lachonospiraceae*. On the basis of its morphological, physiological, chemotaxonomic and phylogenetic properties, we propose the name *Fusimonas intestini* gen. nov., sp. nov. for the mice gut associated strain AJ110941^P^.

**Table 1:** Phenotypic characteristics of strain AJ110941^P^ and its related members in the family *Lachnospiraceae*.

Characteristics	1	2	3	4	5
Isolation source	Feces (Mouse)	Feces (Human)	UASB reactor	Clinical sample (Human)	Blood (Human)
Cell shape	Fusiform	Coccus	Oval/Rod	Rod	Rod
Cell size (μm)	9.0–62.5×0.55–0.9	0.5–1.0	0.5–10.0×0.5–1.0	2.0–5.0×0.8–1.1	3.4–7.3×0.4–0.7
Motility	−	−	+	nd	−
Flagella	+(multiple)	nd^*^	+(one flagellum)	nd	−
Spore formation	−	nd	+	+	−
Biofilm formation (48h)	6.28	2.47	nd	3.84	3.88
(OD_595_) (96h)	20.58	3.46	nd	11.45	4.96
DNA G+C (mol%)	41.1	nd	32	nd	46.0
Growth temperature (°C)	15–40	35–37	20–60	37	15–45
Growth pH	6.5–8.0	nd	6.5–8.0	nd	nd
Major fatty acid	C_18:1_ *cis*9	C_16:0_	nd	nd	C_16:0_
Catalase reaction	−	−	−	nd	+
Utilization of:					
Arabinose	−	−	+	nd	−
Cellobiose	+	−	+	−	−
Fructose	−	+	+	nd	−
Galactose	+	+	−	nd	−
Glucose	+	+	+	+	−
Glycerol	−	−	+	nd	−
Inositol	−	+	+	nd	−
Lactose	+	−	−	−	−
Maltose	+	−	+	+	−
Mannitol	−	+	+	−	−
Mannose	−	−	−	+	−
Melezitose	−	−	nd	−	−
Melibiose	+	−	+	nd	nd
Raffinose	+	−	nd	−	−
Rhamnose	−	−	−	+	−
Ribose	−	+	+	nd	−
Starch	+	−	+	−	−
Sorbitol	−	+	nd	−	−
Sucrose	+	+	+	+	−
Trehalose	+	−	nd	+	−
Xylose	+	−	+	+	−

Strains: 1, AJ110941^P^ (data from this study); 2, *Ruminococcus gauvreauii* CCRI–16110^T^ (Domingo *et al*., 2008^19^); 3, *Clostridium amygdalinum* BR-10^T^ (Parshina *et al*.^20^); 4, *Clostridium citroniae* RAM16102^T^ (Warren *et al*.^21^); 5, *Eisenbergiella tayi* B086562^T^ (Amir *et al*.^11^). ^*^nd, not determined.

### Description of *Fusimonas* gen. nov

*Fusimonas* (Fu.si.mo′nas. L. n. *fusus* spindle; L. fem. n. *monas* a unit; N.L. fem. n. *Fusimonas* a spindle-shaped bacterium (unit)).

Cells are Gram-positive, non-motile, non-spore-forming and long fusiform-shaped. The strain is mesophilic and strictly anaerobic. The DNA G + C content is 41.1 mol%. The main fatty acids are C_18:1_ *cis*9, C_16:0_, and C_18:1_ *trans*9 or *cis*6. The major respiratory quinone is MK-5 (H4). The type species of the genus is *Fusimanas intestini*.

### Description of *Fusimonas intestini* sp. nov

*Fusimonas intestini* (in.tes.ti′ni. L. gen. n. *intestini* of the gut).

The species displays the following characteristics in addition to those given in the genus description. Cells are long fusiform-shaped, approximately 9.0–62.5 µm in length and 0.55–0.9 µm in width. Growth occurs at 15–40 °C (optimum temperature at 37 °C), at pH 6.5–8.0 (optimum growth at pH 8.0), and at 0.3–0.5% (w/v) NaCl. In GAM agar, colonies (0.1–1.4 mm in diameter and <0.2 mm height) are brown-color and disk like form. The strain is catalase-negative. Based on testing with the API ZYM, API ID32A and API 20 A system, naphthol-AS-BI-phosphohydrolase, α-galactosidase, β-glucuronidase, β-glucosidase, α-glucosidase, α-arabinosidase and *N*-acetyl-β-D-glucosaminidase are detected. No activity is found for alkaline phosphatase, esterase, lipase, arylamidase, trypsin, chymotrypsin, acid phosphatase, β-galactosidase, α-mannosidase, α-fucosidase, urease, arginine dihydrolase, glutamate decarboxylase, and protease. The following substrates serve as a sole carbon source for the isolate: cellobiose, glucose, lactose, maltose, raffinose, xylose, sucrose, trehalose, galactose, xylan, starch and melibiose. Arabinose, rhamnose, ribose, mannose, fructose, glycerol, cellulose, pectin, soytone, pyruvate, crotonate, tryptone, casamino acids, yeast extract, H_2_/CO_2_, inositol, mannitol, melezitose and sorbitol do not support growth. The end-products of glucose metabolism are common short chain fatty acids and CO_2_. The cellular fatty acids profile includes C_18:1_ *cis*9 (44.3%), C_16:0_ (22.5%), C_18:1_ *trans*9 or *cis*6 (11.4%), C_14:0_ (9.8%), C_18:0_ (8.5%), C_17:1_ or cycloC_17:0_ (3.5%).

The type strain is AJ110941^P^ isolated from the feces of five weeks-old hyperglycemic obesity model mouse. Strain AJ110941^P^ has been deposited in the International Patent Organism Depositary (IPOD), National Institute of Technology and Evaluation (NITE) as a patent strain (FEMR BP-11443).

## Methods

### Cultivation of strain AJ110941P

Strain AJ110941^P^ (P = patent strain) was isolated from the feces of five weeks-old *db/db* mouse (obesity model mouse) as described previously^18^. In brief, the cultivation and isolation were performed using Eggerth-Gagnon medium (pH 7.7) with headspace gas of H_2_/N_2_/CO_2_ (5:90:5, v/v/v) at 37 °C under anaerobic conditions^28^. Since strain AJ110941^P^ was also able to grow in Gifu anaerobic medium (GAM, Nissui Pharmaceutical Co. Ltd, Tokyo, Japan) with headspace gas of N_2_/CO_2_ (80:20, v/v), liquid and solid (containing 1.6% agar) GAM media were used for further morphological and physiological characterizations.

### Morphological, physiological and biochemical analyses

Cells of strain AJ110941^P^ were observed by phase-contrast microscopy (PROVIS, Olympus, Tokyo, Japan), transmission electron microscopy (H-7000, Hitachi, Tokyo, Japan), and scanning electron microscopy (S-4500, Hitachi) as described previously^29,30^. Gram staining was performed using a Gram-stain kit (Wako, Tokyo, Japan) according to the manufacturer’s instructions, and stained-cells were observed by microscopy. Temperature range for growth were investigated on GAM liquid cultures incubated at 5, 10, 15, 20, 25, 30, 35, 37, 40, 45, and 50 °C, respectively. The pH ranges were tested at pH 5.0, 6.0, 6.5, 7.0, 7.5, 8.0, 8.5, 9.0, and 10.0. The NaCl concentration range for growth was determined at 0.3, 0.5, 1.0, 1.5, 2.0, and 2.5%. The biochemical features were characterized using API ZYM, API ID32A and API 20 A (BioMerieux, SA, France) as described previously^31^. Sole carbon source utilization test was performed using the basal medium supplemented with one of 32 different carbon sources as described previously^32^. The basal medium contained (per liter): KH_2_PO_4_, 0.136 g; NaHCO_3_, 2.52 g; NH_4_Cl, 0.535 g; MgCl_2_.6H_2_H, 0.204 g; CaCl_2_.2H_2_O, 0.147 g; Na_2_S.9H_2_O, 0.3 g; cysteine·HCl, 0.3 g; trace elements solution^32^, 1 ml; vitamin solution^32^, 1 ml; and resazurin solution (1 ml; 1 mg ml^−1^). All cultures were incubated anaerobically at 37 °C. Growth and substrate utilization were determined by monitoring increase in OD_600_. Catalase activity was determined using a 3% hydrogen peroxide solution. Main end-products of glucose metabolism were determined by using LC20 HPLC system with a Shim-pack SPR-H column (Shimadzu, Tokyo, Japan) and GC-2014 gas chromatography (Shimadzu) after 8 days of cultivation.

### Chemotaxonomic analyses

Chemotaxonomic analyses of strain AJ110941^P^ were performed according to the previously described methods^33^. Briefly, the genomic DNA G + C content and major respiratory quinones were analyzed using LC10 HPLC system with a Shim-pack CLC-ODS (Shimadzu) and Zorbax SB-C18 (Agilent Technologies Palo Alto, CA, USA), respectively. Cellular fatty acid compositions were determined using a GCMS-QP2010 system (Shimadzu).

### Phylogenetic analysis based on 16S rRNA gene

Nearly complete 16S rRNA gene sequence (1469 nt, GenBank accession no. AB861470) of strain AJ110941^P^ was previously determined^18^. Comparative 16S rRNA gene sequence analysis was performed using BLAST program against the nucleotide collection (nr/nt) database at NCBI. Multiple alignments of the 16S rRNA gene sequences of strain AJ110941^P^ were performed with its closest relatives within family *Lachnospiraceae* using CLUSTAL W program. The phylogenetic tree was constructed by neighbor-joining method. The percentage nucleotide similarity was calculated by using p-distance available in MEGA software^34^. Bootstrap values were estimated using neighbour-joining and maximum-likelihood methods (each 1,000 replications).

### Biofilm formation assay

We determined the biofilm formation activity for strain AJ110941^P^ and other eight close relatives (type strains) of the family *Lachnospiraceae*; *Anaerostipes caccae* JCM 13470 ^T^ ^22^; *Blautia hydrogenotrophica* DSM 10507 ^T^ ^35^; *Clostridium citroniae* DSM 19261 ^T^ ^21^; *Desulfotomaculum guttoideum* DSM 4024 ^T^ ^24^; *Eisenbergiella tayi* DSM 26961 ^T^ ^11^; *Eubacterium fissicatena* DSM 3598 ^T^ ^36^; *Murimonas intestini* DSM 27577 ^T^ ^15^; *Ruminococcus gauvreauii* JCM 14987 ^T19^. Biofilm formation assay was performed by crystal violet method as described previous study^37^. In brief, full-grown cultures of strain AJ110941^P^ and other relative spices were inoculated to fresh GAM broth (1% inoculation) in 96-well polystyrene tissue culture plates (Becton Dickinson Labware, Franklin Lakes, NJ, USA) in anaerobic glove box. After static incubation for 48 h and 96 h at 37 °C under anaerobic condition, 20 μl of 1% crystal violet solution (solved in 33% acetic acid; Wako, Osaka, Japan) was added. After static incubation for 30 min at room temperature, all crystal violet solutions were removed and each wells was carefully washed twice with sterilized distilled water. The crystal violet stained substances were solved in 95% ethanol, and measured the absorbance of the crystal violet solution on a SPARK 10 M multimode microplate reader (TECAN, Männedorf, Switzerland) at 595 nm. For microscopic observation, biofilm-like aggregate of strain AJ110941^P^ grown in GAM medium for 96 h was transferred to the glass surface (Matsunami, Osaka, Japan), dried, washed with sterilized distilled water, and stained with crystal violet solution. Phase-contrast image was collected by using an Olympus PROVIS microscope.

### Metagenomic database search of 16S rRNA gene sequence of strain AJ110941^P^

The potential habitability of strain AJ110941^P^ was investigated using IMNGS web platform (https://www.imngs.org/)^27^, which is the biggest and most detailed 16S rRNA gene amplicon datasets available to date. The current size of the IMNGS database includes 88,579 of 16S rRNA gene amplicon datasets from 96 different environments^27^. In the present study, we used the 16S rRNA gene sequence of strain AJ110941^P^ (AB861470) as a query sequence, and selected the all 16S rRNA sequences available from the NCBI sequence reads archive (SRA) with 97% sequence similarity threshold. The results of metagenomic database search were categorized based on the level of sequence similarities by BLAST search above 99 and 97% similarities, respectively.

## Electronic supplementary material

Supplementary Information
