# Supplementary material for: Fusimonas intestini gen. nov., sp. nov., a novel intestinal bacterium of the family Lachnospiraceae associated with diabetes in mice
Source: Sci Rep. 2017 Dec 22;7:18087. doi: 10.1038/s41598-017-18122-2 (PMC5741734; doi:10.1038/s41598-017-18122-2)
Supplement: Supplementary file 1 — Supplementary Information [file 41598_2017_18122_MOESM1_ESM.pdf]

Supplementary information:

# *Fusimonas intestini* gen. nov., sp. nov., a novel intestinal bacterium of the family *Lachnospiraceae* associated with diabetes in mice

Hiroyuki Kusada[1§], Keishi Kameyama[2§], Xian-Ying Meng[1], Yoichi Kamagata[1] & Hideyuki Tamaki[1*]

[1.] *Bioproduction Research Institute, National Institute of Advanced Industrial Science and Technology (AIST), Central 6, 1-1-1 Higashi, Tsukuba, Ibaraki 305-8566, Japan*
[2] *Frontier Research Laboratories, Institute for Innovation, Ajinomoto Co., Inc., 1-1 Suzuki-cho, Kawasaki, Kanagawa 210-8681, Japan*

[§]These authors contributed equally to this work.

[*]Correspondence: Hideyuki Tamaki, Bioproduction Research Institute, National Institute of Advanced Industrial Science and Technology (AIST), Central 6-10, Higashi 1-1-1, Tsukuba, Ibaraki 305-8566, Japan
Tel: +81-29-861-6591. Fax: +81-29-861-6587. E-mail: tamaki-hideyuki@aist.go.jp

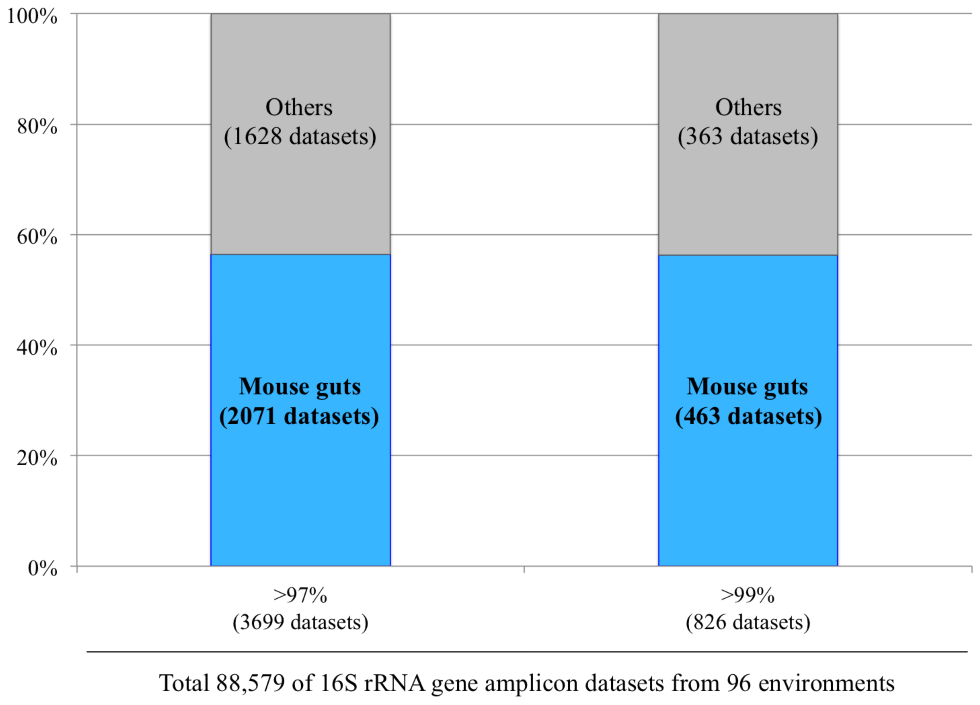


**Supplementary Figure S1. Potential habitability of *F. intestini* strain AJ110941$^{P}$ based on IMNGS search using the 16S rRNA gene sequence (AB861470).** Prevalence of *F. intestini*-related sequences (>97% and >99% sequence similarity) against all 16S rRNA sequence datasets. The relative abundances of *F. intestini*-related 16S rRNA gene sequences in mouse guts and non-mouse guts are shown in blue and gray, respectively.
